# 

**Published:** 1885-06

**Authors:** 


### CONTENTS:

Art. I—Southern Dental Association. Proceedings of the Annual Meeting held in New Orleans, La. in March, 1885 ..... 49

### EDITORIAL, ETC.

American Dental Association ..... 90
Headache ..... 90

### MONTHLY SUMMARY.

Dangers of Wearing Small Dentures ..... 92

An Appetite for Mortar ..... 93

Mortality of Infants ..... 94

Incipient Stages of Inebriety ..... 95

Teeth Without Plates ..... 95

Calcification of the Teeth ..... 96

